# Machine learning for screening and predicting the availability of medications for children: a cross-sectional survey study

**DOI:** 10.3389/fped.2024.1341199

**Published:** 2024-06-18

**Authors:** Jing-yan Guo

**Affiliations:** School of Health Economics and Management, Nanjing University of Chinese Medicine, Nanjing, China

**Keywords:** medications, availability, pediatric, machine learning, XGBoost

## Abstract

**Objective:**

The aim of the study was to explore the factors influencing the availability of medications for children, and establish a machine learning model to provide an empirical basis for the subsequent formulation and improvement of relevant policies.

**Methods:**

Design: Cross-sectional survey. Setting: 12 provinces, China. Medical doctors from 25 public hospitals were enrolled. All data were randomly divided into a training set and a validation set at a ratio of 7:3. Three prediction models, namely random forest (RF), logistic regression (LR), and extreme gradient boosting (XGBoost), were developed and compared. The receiver operating characteristic curve (ROC) and the associated area under the curve (AUC) were used to evaluate the three models. A nomogram and clinical impact curve (CIC) for availability of medication were developed.

**Results:**

Fifteen of 29 factors in the database that were most likely to be selected were considered to establish the prediction model. The XGBoost model (AUC = 0.915) demonstrated better performance than the RF model (AUC = 0.902) and the LR model (AUC = 0.890). According to the Shapley additive explanation values, the five factors that most significantly affected the availability of medications for children in the XGboost model were as follows: the relatively small number of specialized dosage forms for children; unaffordable medications for children; public education on the accessibility and safety of medication for children; uneven distribution of medical resources, leading to insufficient access to medication for children; and years of service as a doctor. The CIC was used to assess the practical applicability of the factor prediction nomogram.

**Conclusions:**

The XGBoost model can be used to establish a prediction model to screen the factors associated with the availability of medications for children. The most important contributing factors to the models were the following: the relatively small number of specialized dosage forms for children; unaffordable medications for children; public education on the accessibility and safety of medication for children; uneven distribution of medical resources, leading to insufficient access to medication for children; and years of service as a doctor.

## Introduction

Medications for children are important for ensuring the level of medical care for children. In the present situation, the availability of medications for children poses a challenge in developing countries (1–3). China is the largest developing country in the world. According to the results of the seventh national population census in China in November 2020, the number of children aged 0–14 years was approximately 250 million, accounting for 17.95% of the total population of China. In the context of an increasing demand for medications, China is also facing a shortage of medication supply for children and a lack of suitable formulations and medication information for children (4).

The availability of medications for children seriously affects the treatment of diseases. Improving the availability of essential medications for children and further promotion of rational medication are the foundations of China's basic medical system. It is also the core function of the national medication policy. Some authors have studied the accessibility of essential medications for children in China (5, 6). However, to the best of our knowledge, there have been no reports on the influencing factors, and a machine learning model to predict medication availability for children has not yet been developed.

In the present study, we surveyed medical doctors with the aim of analyzing the current situation of the availability of medications for children, exploring the factors influencing medication availability for children, and establishing a machine learning model to provide empirical basis for the subsequent formulation and improvement of relevant policies.

## Methods

A cross-sectional study and survey were conducted in China from July 2023 to September 2023. We surveyed medical doctors from the health care system in the region, including tertiary hospitals (comprehensive tertiary first-class hospitals and tertiary children's hospitals) and secondary hospitals.

The survey database included the following factors: type of doctor; hospital level; years of service as a doctor; lack of clear medication guidance and difficulty in diagnosis and treatment; relatively small number of specialized dosage forms for children; public education on the accessibility and safety of medications for children; unaffordable medications for children; lack of drug information (lack of information, leading to unreasonable use of medication); training and education of medical staff on improving the safety of medications for children(Regular training on rational medication for medical staff); establishing a multidisciplinary approach to pediatric medication(Some diseases require multidisciplinary discussions before medication can be administered); parents or patients lacking necessary knowledge about children's medications; insufficient number of pediatricians in the local area; uneven distribution of medical resources, leading to insufficient access to medication for children; unified and standardized standards for pediatric medication; improvement in promoting accessibility to medications for children by the government; establishing a database of rational medication for children; strengthening the selection and allocation management of medications for children; pharmacists providing pediatric pharmacy guidance; registration and regulatory's impact on the accessibility of medications for children; high costs of children's medication research and development; high technical barriers for research; long cycle from research and development to clinical use; difficulty recruiting children for clinical trials; strict safety requirements for children's medications; lower market reward; limited applicable group; higher raw and auxiliary materials; higher taste requirements; and short medication cycle.

### Model development and statistical analysis

In the present study, all data were randomly divided into a training set and a validation set at a ratio of 3:1. The following supervised machine learning methods were used to develop the predictive models, logistic regression (LR), random forest (RF) and eXtreme Gradient Boosting (XGboost). Parameters were adjusted on the training set. During the training process, RF, XGBoost and LR models were initialized separately; then a batch gradient descent algorithm was applied to iteratively update these parameters until convergence was achieved. The receiver operating characteristic curve (ROC) and the associated area under the curve (AUC) were used to evaluate the three models. A nomogram and a clinical impact curve (CIC) for the availability of medications were developed by selecting the 15 most significant predictors from the best model in order to assess the applicability and utility net advantages of the model with the highest diagnostic value.

Statistical analysis was performed with Python (version 3.9) and R software. Non-normally distributed numerical variables were expressed as median and assessed using the Mann–Whitney *U* test. Frequency and percentage were used to present categorical variables, and Pearson's chi-square test was used for intergroup comparison. *P* < 0.05 was considered significant.

## Results

### General information

A total of 154 doctors (123 pediatricians, 17 pediatric surgeons, and 14 from other departments) from 12 provinces in China were enrolled in the survey. Data for 15 out of the 29 variables in the database of this cross-section survey were used in the final study. Table 1 summarizes the general findings of the survey. Among the 154 doctors, 31 were from Class A tertiary hospitals, 119 were from Third Class A children's hospitals, and 4 were from secondary hospitals.

**Table 1:** The general findings of the survey on availability of medication for children.

Variable	Availability (*n* = 60	Un-availability (*n* = 94)	*P*
Doctor type			0.692
1	49	74	
2	7	10	
3	4	10	
Hospital level			0.132
1	12	19	
2	48	71	
3	0	4	
Years of service as a doctor M (P25,P75)	10 (6.7,17)	10 (6,13)	0.445
Lack of clear medication guidance/bringing difficulties in diagnosing and treating (*n*)	56	70	0.002
The relative small number of specialized dosage forms for children (*n*)	47	22	0.000
Public education on the accessibility and safety of medication for children (*n*)	37	62	0.712
Unaffordable medicines for children (*n*)	30	32	0.049
Lack of drug information (*n*)	48	60	0.030
Training and education to medical staff on improving the safety of medication for children( *n*)	54	78	0.215
Establishing a multidisciplinary approach to pediatric medication (*n*)	59	88	0.248
Parents or patients lack necessary knowledge about children medication (*n*)	47	57	0.020
Sufficient pediatricians (*n*)	21	45	0.114
The uneven distribution of medical resources leads to insufficient access to medication for children (*n*)	51	62	0.009
Unified and standardized standards for pediatric medication (*n*)	58	84	0.129
Hospital having standardized medicationstandards for children (*n*)	42	70	0.545
Improvement in promoting accessibility to medication for children by the government (*n*)	58	85	0.204
Establishing a database of rational medication for children (*n*)	33	56	0.575
Strengthen the selection and allocation management of medication for children (*n*)	39	61	0.989
Pharmacists provide pediatric pharmacy guidance (*n*)	43	63	0.543
Registration and regulatory impact on the accessibility of medication for children (*n*)	31	36	0.103
High costs on children medication research and development (*n*)	45	63	0.288
High technical barriers	43	57	0.159
Long cycle from research and development to clinical use (*n*)	43	65	0.739
Difficulty in recruiting children for clinical trial (*n*)	46	65	0.307
Stricter safety requirements for children medication (*n*)	49	68	0.181
Low market reward (*n*)	43	61	0.379
Limited applicable group (*n*)	47	65	0.208
Higher and higher taste requirements of raw and auxiliary materials (*n*)	46	64	0.246
Short medication cycle (*n*)	37	60	0.786

Doctor type, 1 within 5 years, 2 from 5 to 10 years, 3 > 10 years.

Hospital level, 1 comprehensive tertiary first-class hospitals; 2 tertiary children's hospitals; 3 secondary hospitals.

### Prediction model

**Figure 1:**
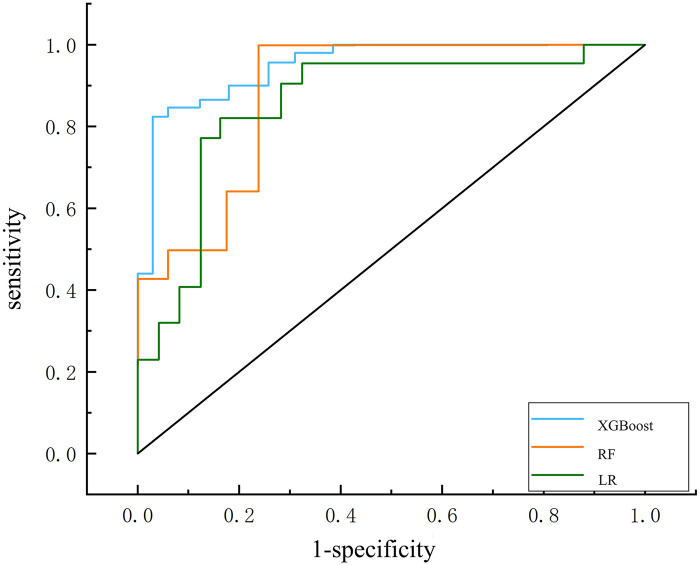
Receiver operating characteristic (ROC) curves of the machine learning classifier in the validation datasets; LR classifier (AUC = 0.890), RF classifier (AUC = 0.902); XGBoost model (AUC = 0.915).

**Figure 2:**
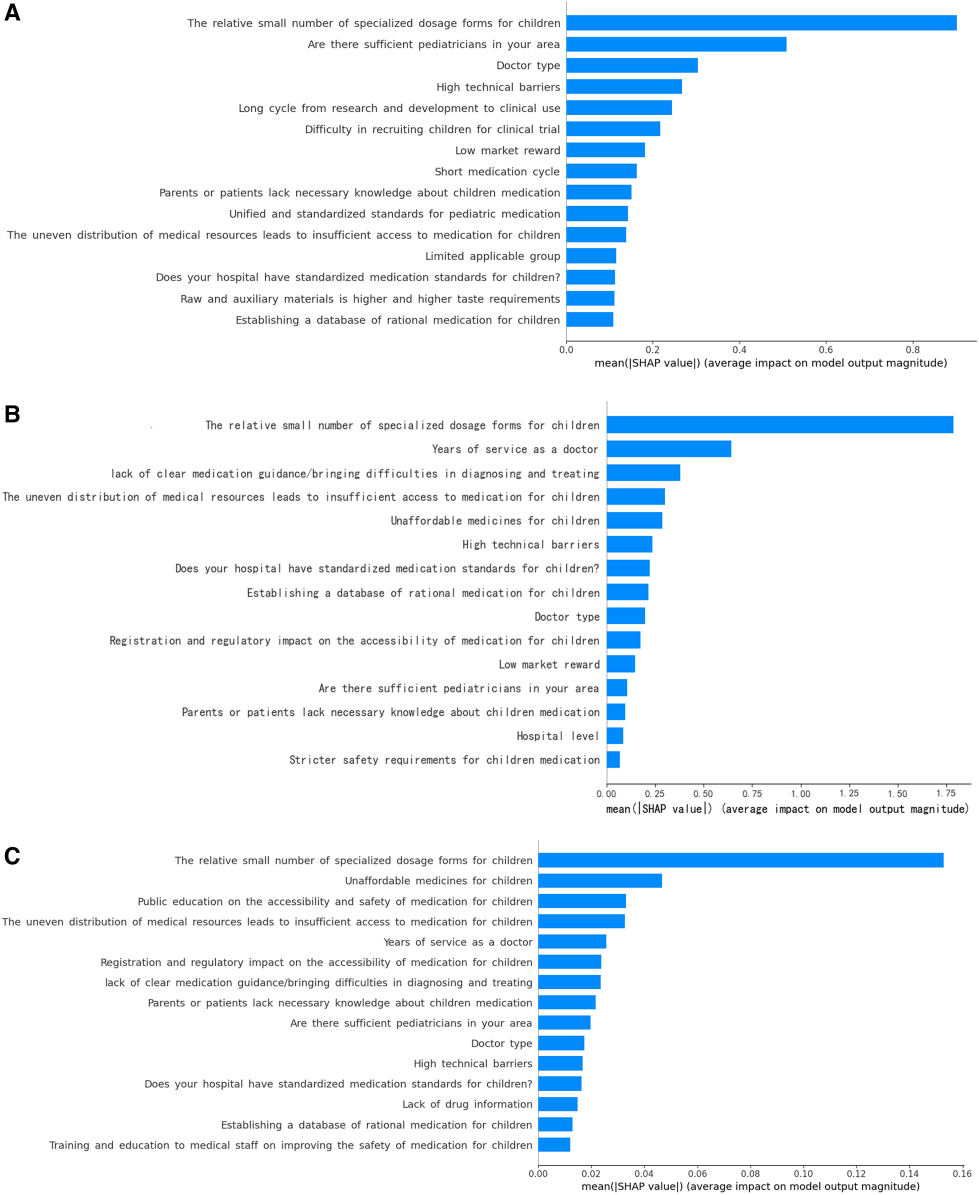
Importance matrix plot of the machine learning models. (**A**) LR model; (**B**) RF model; (**C**) XGBoost model.

Only 15 out of the 29 factors in the database that were most likely to be selected were considered to establish the prediction model. We showed that the XGBoost model (AUC = 0.915) demonstrated better performance than the RF model (AUC = 0.902) and the LR model (AUC = 0.890) (Figure 1 and Table 2). In addition, the XGBoost model showed higher accuracy (0.883) and specificity (0.866) than the LR model (accuracy, 0.828; specificity, 0.825) and the RF model (accuracy, 0.852; specificity, 0.843) in the validation set. Figures 2A–C depicts the relative values of each feature in different models.

**Table 2:** Model performance in the present study.

	LR	RF	XGboost
Accuracy	0.828	0.852	0.883
Sensitivity	0.843	0.861	0.896
Specificity	0.825	0.842	0.866
AUC	0.890 (0.803–0.910)	0.902 (0.852–0.932)	0.915 (0.876–0.965)

**Figure 3:**
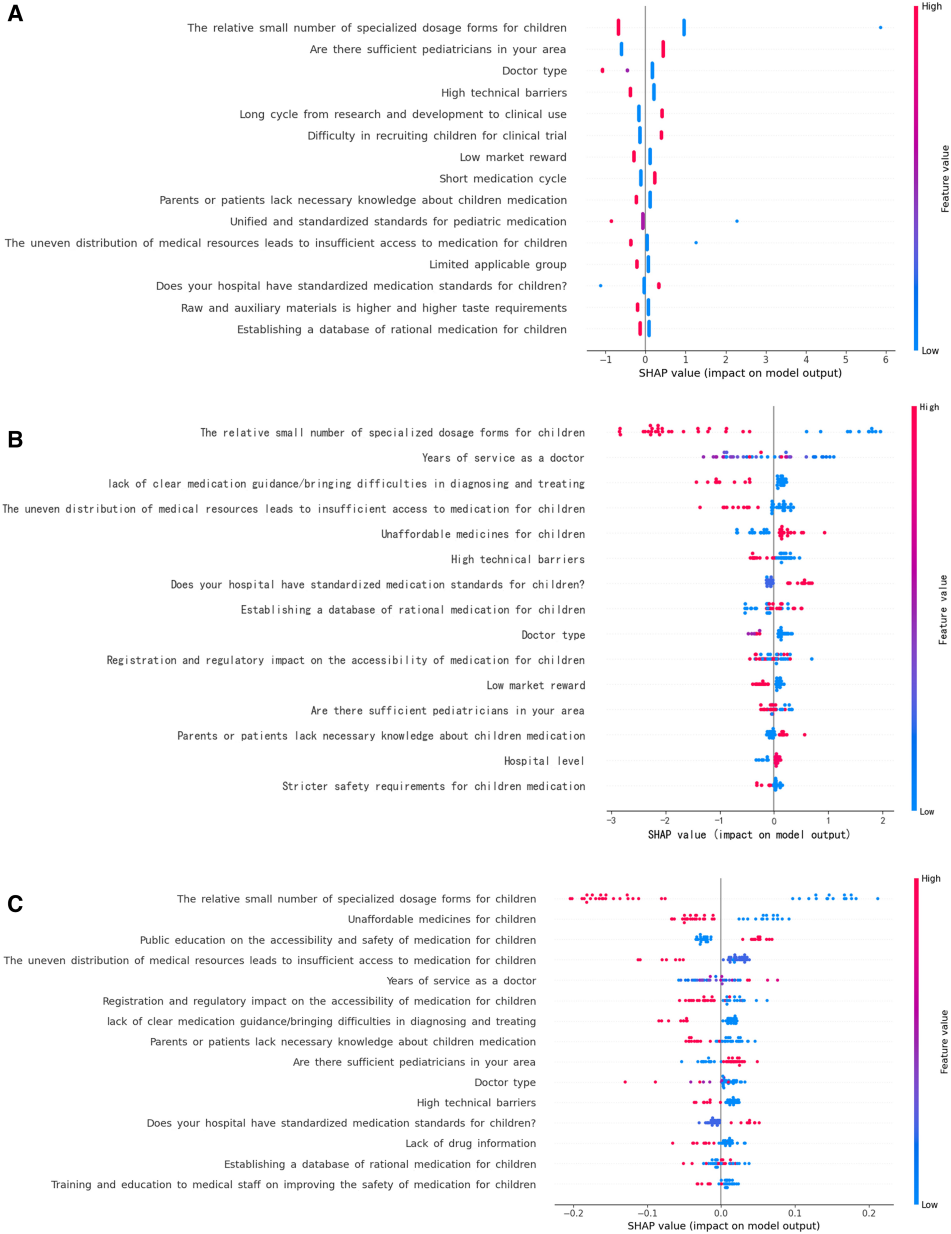
Shapley additive explanations (SHAP) framework for the features in the three machine learning models. (**A**) LR model; (**B**) RF model; (**C**) XGBoost model.

**Figure 4:**
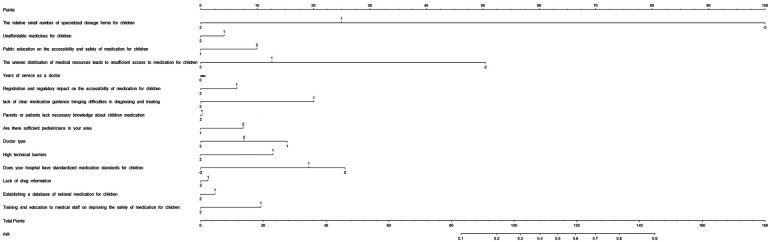
Nomogram to estimate risk factors for the availability of medications. The value of each variable was scored on a point scale from 0 to 100, after which the scores for each variable were added together. That sum was located on the total points axis, which enabled US to predict the probability of the availability of medications for children.

**Figure 5:**
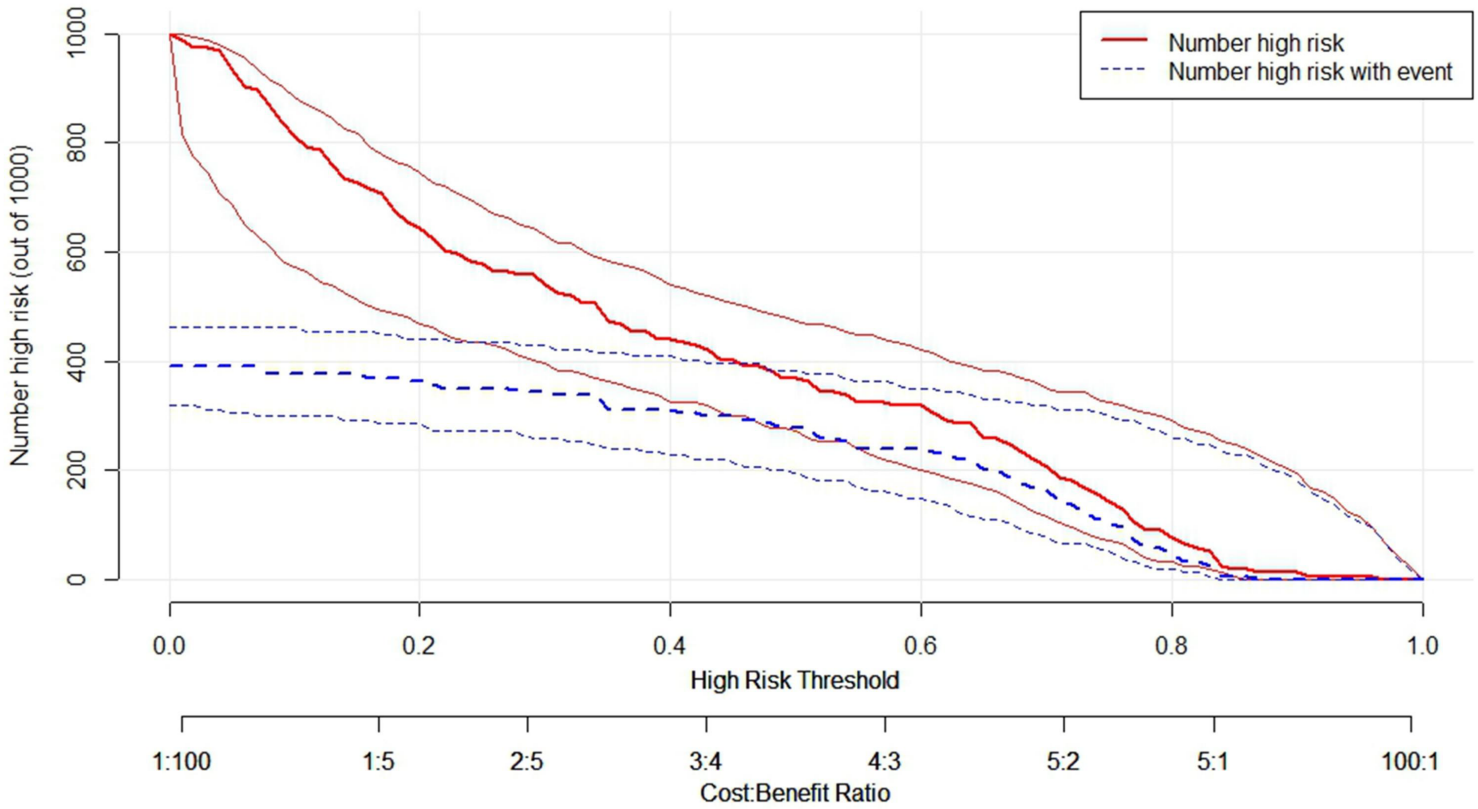
Clinical impact curve (CIC) of XGBoost model, the solid lines (number of high-risk) indicated the number of availability of medications for children who were classified as positive (high risk) by the model at each threshold probability; the dotted lines (number of high-risk events with outcome) was the number of true positives at each threshold probability; CIC visually indicated that nomogram conferred high practical net benefit and confirmed the practical value of the XGBoost model.

Because the XGboost model showed higher accuracy and specificity than the RF and LR models, it was used to establish the risk nomogram. According to the Shapley additive explanation (SHAP) values, the following factors most significantly affected the availability of medications for children in the XGboost model: the relatively small number of specialized dosage forms for children; unaffordable medications for children; public education on the accessibility and safety of medication for children; uneven distribution of medical resources, leading to insufficient access to medication for children; and years of service as a doctor (Figures 3A–C). The 15 most significant factors were integrated to aid in the visualization of the XGboost prediction model. The results indicated that the XGboost model demonstrated reliable prediction of the factors influencing the availability of medications for children in China. Furthermore, we used CIC to assess the practical applicability of the factor prediction nomogram (Figure 4). The results of the CIC analysis indicated that that the nomogram had a superior overall net benefit within the large and practical ranges of threshold probabilities, indicating that the XGboost model has substantial predictive power in practice (Figure 5).

## Discussion

### General findings in the availability and affordability of medications for children

The present study focused on the availability of medications for children. A total of 154 doctors from 12 provinces in China were enrolled in this cross-section survey. The results showed that many factors affected the availability of medications for children. Some factors emphasized the patients’ perspective, such as public education on the accessibility and safety of medications for children; unaffordable medications for children; and parents or patients lacking necessary knowledge about children’s medication. Some factors emphasized the doctors’ perspective, such as years of service; lack of clear medication guidance and difficulties in diagnosis and treatment; shortage of pediatricians; doctor type; and training and education of medical staff on improving the safety of medications for children. Some factors were related to the shortage of resources, and these included the relatively small number of specialized dosage forms for children; registration and regulator's impact on the accessibility of medications for children; the uneven distribution of medical resources, leading to insufficient access to medications for children; high technical barriers; standardized medication standards for children in hospital; and establishing a database of rational medication for children.

### Factors associated with the availability of medications for children

Machine learning tools have been applied in healthcare decision-making (7, 8). In the present study, we used machine learning to establish a model for detecting the factors associated with the availability of medications for children. We used three methods to establish the prediction models, namely RF, LR, and XGBoost. Among the three models, the XGBoost model performed better than the RF and LR models, showing higher accuracy and specificity. The advantage of XGBoost is the presence of random seeds, which improves the model by repeating operations even if the parameters remain unchanged, can efficiently and flexibly handle missing data, and may assemble weak prediction models to generate accurate predictions. Therefore, XGBoost performs better in terms of calculation speed (9). Therefore, we used the XGboost model for further analysis. To the best of our knowledge, these models were the first attempt to establish mathematical models to predict the availability of medications for children.

Access to essential medications for children is challenging, especially in developing countries (1–3). There have been some studies on the availability of essential medications for children using the standardized WHO/HAI methodology (5, 6, 10). Sun et al. reported that the availability of essential medications for children was low in public and private sectors. Specifically, the mean availability of existing generic medications and their original products was less than 40% in both public and private sectors. According to Chen et al., the average availability of essential medications for children in China is 1.6%–46.5% (5). These authors have focused on essential medications from China, and showed that the availability of essential medications in China is a challenge. Thus, we conducted a survey from the doctor's view of point to further explore the potential factors influencing the availability of medication for children.

In the present study, 15 factors that are most likely to be considered in practice were selected to develop the model. According to the XGboost model, the following five factors most significantly affected the availability of medications for children: the relatively small number of specialized dosage forms for children; unaffordable medications for children; public education on the accessibility and safety of medication for children; uneven distribution of medical resources, leading to insufficient access to medication for children; and years of service as a doctor.

In the present study, the relatively small number of specialized dosage forms for children was a key factor affecting the availability of medications for children. As reported by Wang, the list of essential medications for children is still unavailable, and lack of access to pediatric essential medications has been causing growing concerns. Strengths and dosage forms suitable for children, such as oral solutions, are still limited and in short supply in the market (11). Acceptability of medication is crucial for all patients. This is particularly true for children, who have different sensory perception, i.e., taste and texture for oral dosage forms, or pain perception for parenteral forms, which may underlie the refusal of therapy (12–14). Another factor affecting the availability of medications for children is related to the parents. There is limited public education on safety and effectiveness of medications for children. Thus, it is necessary to enhance safety education of the public about medication for children. The issue of unaffordable medication for children is common in developing countries, and China is the largest developing country in the world. The high prices of certain essential medications are still a challenge in some developing areas in China. This point has been reported previously (13). There is uneven distribution of medical resources, leading to insufficient access to medication for children in some aeras in China. There is a shortage of pediatricians in some areas because postgraduates are reluctant to become pediatricians and many pediatricians prefer working in developed regions and large medical institutions. We found that years of service as a doctor also affected the availability of medications for children. From the SHAP values, working experience as a doctor was negatively correlated with the availability of medications for children. Our data imply a vital potential of these measures for further training and establishing of a database of rational medication for children. We also need standardized medication standards for children to improve the availability of medications for children.

Previous studies have indicated that the shortage dose for children, high price, and uneven distribution of medical resources are the main causes of the inaccessibility of medications for children in China (5, 15, 16). All these studies are consistent with the prediction results of the present study. This suggests the feasibility of the prediction models for determining the factors associated with availability of medication for children. The advantage of the present study is that machine learning model was developed and used to screen the factors affecting the inaccessibility of medications for children in China. And nomogram was used to provide the sum of the scores to national policy makers and pharmaceutical management departments for estimating the factors associated with availability of medications for children. CIC visually indicated that nomogram conferred high practical net benefit and confirmed the practical value of the XGBoost model, and thus could help improve the availability of these medications.

### Strengths and limitations

We herein executed a cross-sectional survey with the aim of analyzing the current situation of the availability of medications for children, exploring the factors influencing medication availability for children, and establishing a machine learning model to provide empirical basis for the subsequent formulation and improvement of relevant policies.

The present study had some limitations. First, it included a relatively small number of medical doctors. Second, the cross-section survey only evaluated the doctors’ perspective, which may have introduced selection bias to the prediction models. Namely, the availability of medications for children also depends on production enterprises, hospitals, social security, and drug supervision, et al. Future cross-section surveys should include production enterprises, hospitals, social security, and drug supervision, more large samples survey from different field and more machine learning algorithms so as to more compressively evaluate the availability of medications for children.

## Conclusion

In the present study, we used machine learning to establish three prediction models to screen the factors associated with the availability of medications for children. Also, the visualization of the prediction model and CIC were used to assess the practical applicability of the factor prediction nomogram. The XGBoost model showed the highest sensitivity and specificity. The five factors that most significantly affected the availability of medication for children were as follows: the relatively small number of specialized dosage forms for children; unaffordable medications for children; public education on the accessibility and safety of medication for children; the uneven distribution of medical resources, leading to insufficient access to medication for children; and years of service as a doctor. This is the first report on the use of machine learning to establish prediction models to screen the factors associated with the availability of medications for children.

## Data Availability

The raw data supporting the conclusions of this article will be made available by the authors, without undue reservation.
